# ERRATA

**DOI:** 10.1590/1677-5449.ER1504

**Published:** 2016

No artigo “Tratamento fisioterapêutico vascular para a doença venosa crônica: artigo de revisão”, com número de DOI: http://dx.doi.org/10.1590/1677-5449.003215, publicado no periódico Jornal Vascular Brasileiro, v. 15, n. 1, p. 34-43, na página 34:

**onde se lia**:

“Leila Manuela Santos Soares”

**leia-se**:

“Leila Manuela Soares dos Santos”

