# Supplementary material for: ERRATA
Source: J Vasc Bras. 2016 Oct-Dec;15(4):341. [Article in Portuguese] doi: 10.1590/1677-5449.ER1504 (PMC5829736; doi:10.1590/1677-5449.ER1504)
Supplement: Translation

# Translation: ERRATUM

In the article entitled “Vascular physiotherapy for treatment of chronic venous disease: review article”, DOI: http://dx.doi.org/10.1590/1677-5449.003215, published in Jornal Vascular Brasileiro, v. 15, n. 1, p. 34-43, on page 34,

**where it reads:**

“Leila Manuela Santos Soares”

**it should be read:**

“Leila Manuela Soares dos Santos”
